# Propionibacterium prosthetic joint infection: experience from a retrospective database analysis

**DOI:** 10.1007/s00590-016-1766-y

**Published:** 2016-03-26

**Authors:** Anna Rienmüller, Olivier Borens

**Affiliations:** Orthopedic Septic Surgical Unit, Department of Surgery and Anesthesiology, Lausanne University Hospital, Lausanne, Switzerland; Department of Orthopedic Surgery, Vienna General Hospital, Medical University Vienna, Waehriger Guertel 18-20, 1090 Vienna, Austria

**Keywords:** Implant, Infection, Biofilm, *Propionibacterium acnes*

## Abstract

**Background:**

With improved diagnostic methods and longer prosthesis indwelling time, the frequency of diagnosed *Propionibacterium* prosthetic joint infections (PJI) is increasing. Data on clinical, microbiological, radiological and surgical treatment are limited, and importance of this organism in PJI is probably underestimated.

**Materials and methods:**

We retrospectively analyzed patients with PJI caused by *Propionibacterium* spp. diagnosed at our institution between 2000 and 2012. Patient data were retrieved through chart review, and the outcome was evaluated at patient follow-up visits.

**Results:**

Of 15 included patients (median age 65 years, range 44–87), 8 hip, 4 shoulder, 2 knee and 1 ankle PJI were recorded. The median time from implantation to diagnosis of PJI was 44.2 months (range 2–180 months). Most PJI (8 patients, 53 %) were diagnosed late (>24 months after arthroplasty). Persistent pain was present in 13, local joint symptoms in 8, fever in 4 and sinus tract in 3 patients. Radiological signs of loosening were present in 11 patients (73 %). Organisms were detected in intraoperative biopsy (*n* = 5), sonication (*n* = 4) or preoperative joint puncture (*n* = 4). In three cases coinfection with a coagulase-negative staphylococcus was diagnosed. Revision surgery was performed in all cases. After a mean follow-up of 16 months after revision surgery (range 4–37 months), 14 patients (93 %) showed no signs or symptoms of infection and had a functional prosthesis; one patient experienced a new infection with another organism (*Staphylococcus epidermidis*).

**Conclusion:**

Patients with persistent postoperative pain and/or loosening of implants should be screened for PJI with low-virulent organisms such as *Propionibacterium*, including.

## Introduction

*Propionibacterium* spp. is an anaerobic Gram-positive rod-shaped bacterium, which is commonly found in the pilosebaceous follicles of the human skin [1], oral cavity, conjunctiva [2], respiratory and intestinal tract [3] and external ear canal [1]. Although it is often considered not pathogenic, *Propionibacterium* spp. can be responsible for severe infections including endocarditis [4], meningitis and brain abscess [5], endophthalmitis [6] conjunctivitis [7] and osteomyelitis or spondylodiscitis [8]. *Propionibacterium* spp. was also isolated in atherosclerotic lesions [9] and several inflammatory conditions, but its pathogenetic role in these clinical situations is less clear.

The capability of forming a biofilm on any kind of implant in the body is a predisposing factor for infection especially in relation to artificial heart valves, ventriculo-peritoneal shunts and orthopedic implants such as joint prosthesis [10]. Although perioperative antibiotic prophylaxis, new implant design and improvement of surgical technique and operating room environment have considerably reduced the risk of intraoperative infection, the frequency of PJI is continuously rising as the number of implanted prosthesis and indwelling time rises, as well as the diagnostic procedures improve, such as sonication of removed prosthesis and molecular diagnostic [11, 12].

*Propionibacterium* PJI typically presents as chronic prosthetic joint infection (PJI) manifesting several months after surgery, rarely as acute postoperative infection [10, 13]. Data on *Propionibacterium* PJI are limited, predominantly originating from case reports or smaller case series. We therefore performed a retrospective cohort study investigating the epidemiology, clinical characteristics, diagnostic pathway, treatment and outcome of patients with PJI caused by *Propionibacterium* spp.

## Patients and methods

### Hospital setting

The study was conducted at the University Hospital of Lausanne in Switzerland, a primary and tertiary healthcare center. It is the major provider of acute medical care for about 300,000 inhabitants. The local institutional review and ethical board approved the study.

### Study population

From January 1, 2000 through December 31, 2012, all episodes of *Propionibacterium* PJI in patients aged ≥18 years were included. Episodes of PJI were identified using the microbiology database and the infectious diseases consultation list. Each episode was evaluated by an orthopedic surgeon and infectious diseases specialist according to predefined criteria (see below).

### Definitions

PJI was defined when at least one of the following criteria was present [14]: (1) visible purulence of a preoperative aspirate or intraoperative periprosthetic tissue (as determined by the surgeon), (2) presence of a sinus tract communicating with the prosthesis, (3) acute inflammation in intraoperative permanent tissue sections by histopathology (as determined by the pathologist), (4) microbial growth in at least two intraoperative periprosthetic tissue samples or in preoperative joint aspirate (and if available synovial fluid with >1700 leukocytes/µl or >65 % granulocytes for knee prosthesis [15], >4200 leukocytes/µl or >70 % granulocytes for hip prosthesis [16], respectively), (5) sonication fluid of the removed implant (>50 CFU/ml). Time to infection was defined as the interval from arthroplasty (or last surgical intervention of prosthesis) to the diagnosis of PJI. According to the time of infection, infections were classified in early (<3 months after surgery), delayed (3–24 months after surgery) and late (>24 months after surgery) [17].

### Data collection

Hospital charts were reviewed with a standardized case report form to retrieve demographic, clinical, and laboratory data. The following data were extracted: age; sex; underlying joint condition; and prosthesis type, date of first insertion, synovial fluid gram stain and culture results of synovial fluid, antimicrobial therapy and treatment outcome. We assessed radiological images at time of diagnosis for signs of loosening (defined as a line zone greater than one mm on one or both components, or for knee prosthesis on one side of the tibial component).

## Results

### Demographics and prosthesis characteristics

Table 1 shows the demographic and prosthesis characteristics of 15 episodes of PJI, including 8 hip, 4 shoulder, 2 knee and 1 ankle PJI. The median age at time of PJI diagnosis was 65 years (range 44–87 years). Primary reasons for arthroplasty were osteoarthritis in 9, post-traumatic arthritis in 4 and hip dysplasia in 2 patients. The median time from implantation to diagnosis of PJI was 44.2 months (range 2–180 months). One patient presented early (<3 months after surgery), 6 were diagnosed delayed (3–24 months after surgery) and 8 late (>24 months after surgery) (see Table 2).

**Table 1:** Demographics and prosthesis characteristics

Characteristics	(*n* = 15)
Age, median (range) [years]	65 (44–87)
Female sex	4 (25)
Length of hospital stay, median (range) [days]	42 (10–246)
*Type of prosthesis*	
Total hip arthroplasty	8 (53.3)
Total knee arthroplasty	2 (13.3)
Total shoulder arthroplasty	2 (13.3)
Partial shoulder arthroplasty	2 (13.3)
Total ankle arthroplasty	1 (6.7)
*Reason for primary arthroplasty*	
Osteoarthritis	8 (53.3)
Trauma	5 (33.3)
Dysplasia	2 (13.3)
*Surgical treatment*	
One-stage exchange	5 (33.3)
Debridement and partial one-stage exchange	2 (13.3)
Two-stage exchange	5 (33.3)
Removal of implant and definitive spacer implantation	2 (13.3)
Arthrodesis	1 (6.7)

Data are no. (%) of episodes, unless otherwise indicated

**Table 2:** Diagnostic steps and classification of infection

Characteristics	(*n* = 15)
Diagnostic steps	
Joint puncture positive for *Propionibacterium* spp	3/8
Biopsy positive	12/15
Sonication positive	3/3
Clinical signs and symptoms	
Pain	13 (86.7)
Pseudoparalysis (shoulder)	2 (13.3)
Fever/sepsis	5 (33.3)
Swelling/joint effusion	9 (60)
Fistula	3 (20)
Radiological loosening	11 (66.7)
CRP (mg/l) < 8	8 (53.3)
CRP (mg/l) > 30	6 (40)
Pre-revision surgery diagnosis	
Propioni prosthetic joint infection	3(20)
Prosthetic joint infection other^a^	3 (20)
Suspicion for infection/loosening^b^	5 (33.3)
Mechanical loosening	6 (40)
Classification of Infection	
Early (<3 months after surgery)	5 (33.3)
Delayed (3–24 months after surgery)	2 (13.3)
Late (>24 months after surgery)	5 (33.3)

Data are no. (%) of episodes, unless otherwise indicated

^a^Co-infection of Propionibacterium spp and

^b^Two patients with partial shoulder arthroplasty were revised for Pseudoparalysis

In three cases, correct diagnosis was known before revision surgery. Three patients with CRP > 30 mg/l were diagnosed for co-infection with coagulase-negative staphylococcus, one patient presented sepsis with bilateral infection of total hip prosthesis

### Clinical characteristics

Most *Propionibacterium* spp. infections were diagnosed as delayed (53 %) or late PJI (40 %) with consistent pain as primary clinical parameter (*n* = 13) followed by radiological loosening (*n* = 11) and persistent sinus tract (*n* = 3) and unspecific clinical signs of infection (*n* = 6). Between onset of symptoms and correct diagnosis of PJI with *Propionibacterium* spp., we found a mean delay of 17 months (range 5 days to 60 months). Seven patients developed infection after primary implantation of joint prosthesis. Three patients underwent surgical debridement and PE-exchange for early infection after primary arthroplasty, and in two patients revision surgery was performed for periprosthetic fracture before onset of symptoms of infection. Two patients underwent multiple interventions (revision prosthesis and debridement) for infection before diagnosis of PJI with *Propionibacterium spp.,* and five patients presented previous PJI with another bacterium. The main symptom at diagnosis were persistent pain in 13, local symptoms (joint swelling, redness or effusion) in 8, fever in 4 and sinus tract in 3 patients. Radiological loosening was present in 11 cases (73 %). Three of four patients with total shoulder arthroplasty presented with pseudoparalysis. CRP values at time point of diagnosis or pre-revision surgery were <9 ml/l in 8 patients, and between 9 and 20 mg/l in 3 infected prostheses. In three patients presenting co-infection with coagulase-negative staphylococcus, we found a CRP higher than 20 mg/l, and in one patient presenting septic infection involving both hip prostheses a CRP of 110 mg/l (see Table 2).

### Microbiology

Co-infection was present in 3 cases with *coagulase*-*negative staphylococcus.* Organisms were detected in preoperative joint puncture only in 3 cases, 6 times joint puncture revealed negative, although we use culture incubation time of 14 days in general. *Propionibacterium spp* infection was confirmed in 5 cases by perioperative biopsy as a first step (Table 2). Since 2006, we additionally used sonication of implants for detection of infection. We could confirm infection with *Propionibacterium* spp 4 times using sonication of implants, although preoperative joint puncture was negative in 3 cases before explantation and intraoperative biopsy was positive only in one sample for one of those cases but CRP values were slightly elevated in two of those cases (12 and 16 mg/l).

### Surgical procedures

All 15 patients underwent surgical intervention. In 5 patients, a one-stage exchange was performed, of whom the pathogen was known preoperatively through joint aspiration (*n* = 1) or revision surgery was done for aseptic/mechanical loosening and correct diagnosis was found using sonication of implants (*n* = 3). Two patients underwent a one-stage exchange of the cup and polyethylene inlay of total hip prosthesis. In a 75-year-old man, revision of TSA was performed for clinical diagnosis of pseudoparalysis with implantation of reversed TSA. *Propionibacterium spp* infection was confirmed in 3/3 biopsies. Two-stage exchange with spacer implantation was performed in 6 cases, definitive spacer implantation in 2 cases and arthrodesis in 1 case (see Table 1).

Duration of hospitalization was 42 days (range 10–246 days); mean time of spacer implantation was 53 days (range 23–98). Antimicrobial treatment included intravenous treatment for at least 2 weeks, followed by per-oral treatment for a total duration of minimum of 6–12 weeks. The unit of infectiology of our hospital, specialized in bone and joint infections, was consulted for diagnosis and treatment of PJI. Since 2006, we follow the algorithm by Zimmerli et al. [14] for treatment of PJI; antibiotics are adapted to the antibiogramm.

### Outcome evaluation

All patients were observed regularly at follow-up visits with a minimum follow-up of 6 months and a maximum of 35 months post-reimplantation (mean follow-up of 14 months). Evaluation of clinical signs and symptoms of infection, C-reactive protein levels and X-ray analysis was performed 3 months after termination of antimicrobial treatment and at scheduled later follow-ups. In 13 patients, CRP values at 6-month follow-up were found to be in the normal range <9 mg/l and no radiological signs of loosening were present. In one patient, a 78-year-old man, initially partially revised (1-step exchange of the cup component only) for suspicion of aseptic loosening, we found increasing CRP values (>30 mg/l) at 6-month follow-up with increasing pain in the thigh while walking. A 65-years-old man needed treatment for new infection of THA with another organism (*Staphylococcus epidermidis*) 6 weeks after revision surgery.

## Discussion

*Propionibacterium* spp. are anaerobic diphtheroids, generally considered as nonpathogenic. In their planktonic form, they are sensible to the host’s immune response and antibiotic treatment. But in contact with implants such as a prosthetic joint they will immediately form an extracellular matrix, the so-called biofilm. Well protected underneath this sheath of biofilm they won’t be reached by the immune system and most antibiotics (except for rifampicin) are not able to penetrate the biofilm [14]. Biofilms are probably present in more than 65 % of bacterial infections with approximately 2 % infection rate for prosthetic joint replacements [18]. Although the number of biofilm-associated infections is constantly rising, little information is available for guidance in diagnostic steps and management for successful diagnosis and treatment of PJI with *Propionibacterium spp*. Establishing the correct diagnosis is of particular importance because slow growth rate and low virulence are delaying presentation of specific signs of infection [19]. Average latency between infection during surgery and clinical manifestation is 4–5 months (=delayed infection) but can extend to up to 13 years post-implantation [20, 21]. In our study, we found latency between surgery and clinical manifestation of infection between 2 months and 15 years after the last surgical intervention. But average time span between onset of clinical symptoms and confirmation of infection/isolation of causative organism was 17 months with a range between 5 days and 5 years. Sixty percent of patients included in our study presented with unspecific signs and symptoms such as chronic pain, joint effusion or decreased mobility already since primary implantation of prosthesis. This underlines the difficulty and also importance of differentiating between “normal” postoperative pain and/or limited function and “low-grade” infection. To correctly diagnose so-called “low-grade” PJI is representing a challenging problem, as there is no single diagnostic modality with absolute sensitivity and specificity [22]. First line investigation is represented by serologic tests, such as measurement of white blood cell count and CRP. They generally have good sensitivity and lower specificity. In presence of PJI with *Propionibacterium spp*, both parameters are often not or little elevated [23, 24] and therefore not helpful in confirming or rejecting diagnosis of infection [24, 25]. In our study, we revealed lower CRP levels in the absence of co-infection or sepsis. Additional information is available from standard radiography showing signs of radiolucency or a positive leukocyte skeletal scintigraphy. But loosening for mechanical reason or infection cannot be confirmed nor excluded, and in our study six patients underwent revision surgery for diagnosis of mechanical loosening. In those patients, joint puncture was negative and diagnosis was confirmed by intra-operative biopsy in 4 cases and by sonication only in two, where intraoperative biopsy also was negative. In general, we can say that aspiration of the joint has high specificity [22], but *Propionibacterium*, as a facultative anaerobic rod is difficult to culture and identify, needing anaerobic culture media and incubation periods of 7–15 days and even intraoperative cultures, although considered as the gold standard, may be negative for some patients with clinical proven prosthetic infection [22]. So accurate diagnosis often requires the use of combinations of tests and a strong clinical suspicion. The patients will mainly present non-specific clinical signs, such as pain, swelling and functional impairment [13]. All patients included in our study presented pain as well as swelling and/or intra-articular fluid in the area of the infected arthroplasty as main symptoms. Fever was only present in case of sepsis or co-infection. More than half of patients included had undergone at least one revision surgery before diagnosis of PJI with *Propionibacterium spp.* Also Zappe et al. [24] could confirm previous prosthetic infection as risk factor for PJI with *Propionibacterium acnes*.

Our study has several limitations. First of all, our results are based on a retrospective analysis of a database of a large reference medical center that doesn’t only include local patients but also referral care for specialized treatment. This might lead to bias, especially in relation to delay of correct diagnosis as many patients have been treated for unspecific symptoms or have been revised for different reason elsewhere before referral to our institution. 53.3 % of PJI had undergone previous revision surgery or partial exchange of implants for suspected infection in relation to unspecific symptoms. But diagnosis of *Propionibacterium spp* infection never could have been confirmed. We didn’t provide diagnostic steps and treatment before referral to our institution. Furthermore, all PJI included represent a heterogeneous group as we included all prosthetic joint infections involving hips, knees, shoulders and ankle joint and not only a single joint type. *Propionibacterium spp* infection is mainly described for PJI of the shoulder [19, 26], but can also occur as delayed or late infection in any other joint replacement or spinal surgery [23, 25]. Additionally, our sample size of confirmed PJI with *Propionibacterium* spp is small, thus limiting statistic power and evaluation.

Another drawback is that diagnostic steps as mentioned above were performed individually, meaning that not every patient underwent joint puncture before revision surgery and anaerobic media for culture was not always used. Inclusion criteria and definition of infection followed Zimmerli et al. [14]; additionally, we decided to also take into consideration positive sonication of implants for *Propionibacterium* spp. after removal. It is important to mention that 4 PJI (27 %) were only confirmed by sonication of implants and could not be diagnosed in preoperative joint puncture or intraoperative biopsy (according to definition of two positive cultures necessary). According to the Philadelphia consensus meeting in 2013 [27], one of those patients would not be included as PJI as he only presented with positive sonication of implants for *Propionibacterium* spp.; this shoulder prosthesis had undergone several revision surgery for instability and pain elsewhere and was revised for persistent pain and instability at our institution. No preoperative puncture was available, and no perioperative culture or tissue sample was evaluated, and CRP was never above 6 mg/l. The preoperative shoulder X-ray suspected loosening of the stem. In relation to the history of the patient and clinical/radiological symptoms, we still considered the prosthesis as infected after sonication and treated accordingly. Already Tunney et al. [28] were able to show that only using bacteria cultures and tissue samples would lead to misdiagnosis of non-infection and not appropriate postoperative treatment. In particular, the proportion of, by additional sonication isolated, *Propionibacterium* spp. was higher than previously reported. We can therefore confirm the statement of Bereza et al. [29] that lack of clinical signs of infection and negative pre- and postoperative cultures do not exclude the presence of microorganism and PJI should be considered in all revisions performed within 2 years of implantation [30]. This shows even more how important it is to establish a standardized diagnostic pathway for identifying germs, causing delayed and/or late infection such as *Propionibacterium spp.,* pre-revision surgery. We suggest that once diagnosis of PJI with *Propionibacterium spp*. is confirmed, consequent treatment should carefully be considered.
